# Observations on Homœopathy and Animal Magnetism, &c.

**Published:** 1839-04

**Authors:** 


Art. XV.—*Observations on Homœopathy and Animal Magnetism, as illustrating the necessity for the employment of Caution in pronouncing a Judgment upon assumed Discoveries in Medical Science; forming a Lecture, introductory to a Course on the Practice of Medicine, delivered at the Royal School of Medicine and Surgery, Pine-street, Manchester, October* 3, 1838. By James Lomax Bardsley, m.d., f.l.s.—*Manchester*, 1838. 8vo. pp. 31.

This lecture contains, as might be expected from its highly respectable and accomplished author, a temperate and able statement of the two chief medical delusions of our time, Hahnemannism and Mesmerism revived. The first of these makes so little progress, and the second is so fully treated of in our present Number, that any remarks upon either would be superfluous; but we can justly congratulate the Manchester medical students on possessing so judicious and reasonable an instructor as Dr. James Bardsley, who can distinguish truth from error, and combine great zeal in the cultivation of practical medicine with the exercise of a sound judgment concerning pretended discoveries in it.

